# Contemporary Management of Renal Cell Carcinoma: A Review for General Practitioners in Oncology

**DOI:** 10.3390/curroncol31080359

**Published:** 2024-08-22

**Authors:** Anish Tejura, Ricardo Fernandes, Stacey Hubay, Matthew Scott Ernst, Mario Valdes, Anupam Batra

**Affiliations:** 1 Division of Medical Oncology, Department of Oncology, Schulich School of Medicine & Dentistry, Western University, London, ON N6A 3K7, Canada; anish.tejura@lhsc.on.ca (A.T.); ricardo.fernandes@lhsc.on.ca (R.F.); 2 Verspeeten Family Cancer Centre, Victoria Hospital, London Health Sciences Centre, London, ON N6A 5W9, Canada; 3 Department of Oncology, Grand River Regional Cancer Centre, 835 King St. W., Kitchener, ON N2G 1G3, Canada; stacey.hubay@grhosp.on.ca (S.H.); matthew.ernst@grhosp.on.ca (M.S.E.); mario.valdes@grhosp.on.ca (M.V.)

**Keywords:** renal cell carcinoma, immunotherapy, checkpoint inhibitor, vascular endothelial growth factor, tyrosine kinase inhibitor, hypoxia-inducible factor, von Hippel Lindau, theranostics

## Abstract

Renal cell carcinoma accounts for a significant proportion of cancer diagnoses in Canadians. Over the past several years, the management of renal cell cancers has undergone rapid changes in all prognostic risk categories, resulting in improved oncologic outcomes. Novel strategies for metastatic disease make use of the synergy between checkpoints and angiogenesis inhibition. Moreover, combination checkpoint inhibition has demonstrated durable efficacy in some patients. Adjuvant immunotherapy has recently shown a survival benefit for the first time in select cases. Significant efforts are underway to explore new compounds or combinations for later-line diseases, such as inhibitors of hypoxia-inducible factors and radiolabeled biomolecules targeting tumor antigens within the neoplastic microenvironment for precise payload delivery. In this manuscript, we provide a comprehensive review of the available data addressing key therapeutic areas pertaining to systemic therapy for metastatic and localized disease, review the most relevant prognostic tools, describe local therapies and management of CNS disease, and discuss practice-changing trials currently underway. Finally, we focus on some of the practical aspects for general practitioners in oncology caring for patients with renal cell carcinoma.

## 1. Introduction

Renal cell carcinoma [RCC] is currently the 10th most common cancer diagnosed in Canada [1]. According to the latest Canadian Cancer Statistics published in 2023, it is estimated that RCC will make up 4.5% of all new cancers diagnosed in men and 2.6% in women [2]. 5-year overall survival [OS] in all-comers is estimated to be 76% [US data from 2009 to 2015], and OS is largely dependent on the stage at initial diagnosis. In patients with stage I disease, the 5-year OS is 93%; for stage II/III, it is 72.5%; and for metastatic disease, it is 12%. In addition, the incidence of RCC is projected to increase over the next several years. This is related to advances in imaging techniques that can detect more incidental masses as well as lifestyle-related factors [3]. The use of immunotherapy and vascular endothelial growth factor tyrosine kinase inhibitors (VEGF TKIs) has rapidly changed the treatment paradigm for RCC. Furthermore, current recommendations incorporate systemic therapy into the management of RCC in an adjuvant setting. Given the increase in the incidence of RCC, the morbidity associated with metastatic disease, and the rapidly changing guidelines for the management of early-stage disease, the purpose of the current article is to review the latest literature on the management of RCC. We aim to focus this article on general practitioners in oncology managing patients with RCC in day-to-day practice.

Renal cell carcinoma has several different pathologic variants. The latest WHO Classification System identifies 21 different histologic sub-types of RCC [4]. The most common type is clear cell renal cell carcinoma (ccRCC), which accounts for approximately 70% of all adult renal epithelial neoplasms. Other common histopathological sub-types include papillary (10–15%) and chromophobe histologies (4–6%) [5]. The focus of the current paper will be on the management of ccRCC. We will review the use of adjuvant therapy in ccRCC, followed by first-line treatment for metastatic disease, discuss subsequent lines of therapy, and explore future therapeutics currently under study that are expected to be practice-changing.

### Methods

A comprehensive literature search was conducted on PUBMED to identify relevant, practicing changing phase III randomized clinical trials as well as prospective and retrospective studies related to the management of RCC. Additionally, the authors also included a search for relevant clinical guidelines from the Canadian Cancer Society and the European Society for Medical Oncology. Keywords such as “Renal Cell Carcinoma” “Management”, “Adjuvant therapy” and “Metastatic disease” were used. Boolean operators were used to narrow the search results. The search was limited to articles published between 1998 and 2024.

Studies were included in the summary if they were focused on the management of renal cell carcinoma, published in a peer-reviewed journal, and were determined to be practice-changing by the authors. A full–text copy of these articles was obtained, and a synthesis of this data was conducted and presented herein.

## 2. Adjuvant Systemic Therapy for RCC

For patients with neoplasms confined to the kidney, surgical resection with nephrectomy or partial nephrectomy of the affected kidney is a curative treatment. In a select group of patients, active surveillance, local ablative therapy, or radiotherapy may be reasonable alternatives to surgical resection [6]. The risk of recurrence following nephrectomy is dependent on both TNM staging and histopathological features. In a cohort analysis of 3651 patients, 29.8% of all-comers had disease recurrence at 9 years of follow-up [7]. Most recurrences are detected within the first two years post-nephrectomy; however, as many as 6% of patients will have recurrences 10 years after their initial surgery [8]. Following nephrectomy, patients are often followed up by their surgical team with serial computed tomography on an active surveillance schedule. However, a study evaluating the efficacy of screening recommendations set out by the National Comprehensive Cancer Network demonstrated that these protocols could miss approximately one-third of recurrences [8]. The goal of adjuvant treatment is to reduce the risk of metastatic disease and improve patient survival.

One of the challenges in the adjuvant setting is appropriately identifying patients who are at the highest risk of recurrence and would benefit the most from systemic therapy. Pre-selection of patients is important to avoid over-treatment for patients at a low risk of relapse. Prognostic models have traditionally been used by urologists to help stratify risk for patients post-nephrectomy. The goal of these models is to help guide surveillance strategies. These include the Leibovich [9], SSIGN [10], and MSKCC models [11] (see references for full information on these models). However, there are conflicting data on which of these models is the most accurate. In addition, subsequent validation studies have shown that these models are not as accurate as originally estimated [12]. The Canadian Urological Society [6] recommends that tumor size, tumor grade, presence of nodal metastasis, and histological subtypes are all important prognostic features in determining the risk of recurrence. Many trials conducted in the adjuvant setting utilize these prognostic features in categorizing patients at a high risk of recurrence.

In the past, several studies have attempted to identify beneficial adjuvant therapies, unsuccessfully. These studies evaluated the efficacy and safety of TKIs that are commonly used in the adjuvant metastatic setting. For example, the phase III ASSURE trial [13] assessed the benefit of one year of treatment with sunitinib, sorafenib, or placebo for 1943 patients from cancer centers in the USA and Canada. The inclusion criteria in this study were pT1b and grade 3–4 disease or any pT2-4 or node positivity. There was a trend favoring sunitinib compared to placebo in improving disease-free survival (5.8 years versus. 6.6 years; 1.02 [CI 0.85–1.23]), but this failed to reach statistical significance. The S-TRAC trial demonstrated a modest disease-free survival benefit for sunitinib in the adjuvant setting (5.6 years versus 6.8 years, HR: 0.76, CI [0.59–0.98], *p* = 0.03) [14]. The main challenge with this strategy was the high incidence of significant toxicities, e.g., the rate of grade ≥3 adverse events was 60.5% in the sunitinib group and 19.4% in the placebo group. Furthermore, subsequent follow-up data did not demonstrate an OS benefit [15]. As a result, it has not been widely adopted in clinical practice and has not been approved by Health Canada.

**Figure 1:**
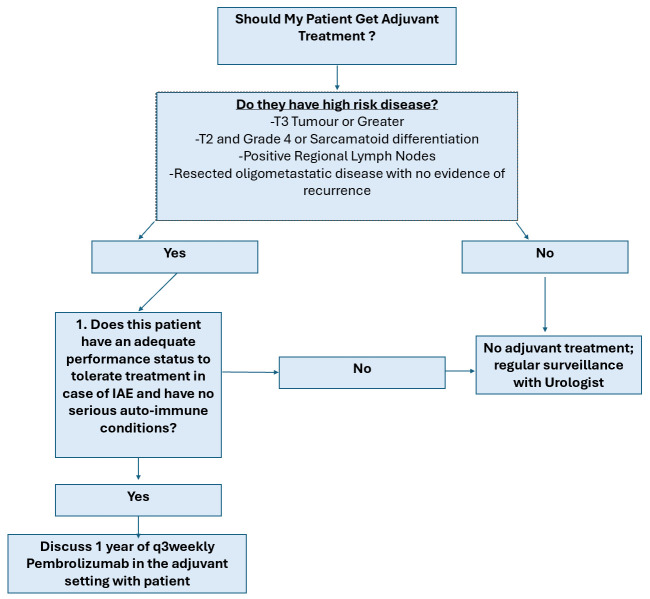
Proposed decision-making algorithm for selection of patients suitable for adjuvant immunotherapy.

An emerging area of interest in adjuvant therapy is the use of immunotherapy. The KEYNOTE-564 trial [16] was the first to demonstrate a benefit in disease-free survival. In the trial, 994 patients who were defined as having a high risk of recurrence (patients with T2 disease with grade 4 differentiation or sarcomatoid differentiation, patients with tumor stage 3 or greater, regional lymphnode metastasis, or patients with the resected metastatic disease within 1 year from initial nephrectomy with no evidence of recurrence) were randomized to one year of pembrolizumab (PD-1 inhibitor) versus placebo. The risk of disease recurrence or death was 32% lower with adjuvant pembrolizumab therapy than with placebo (hazard ratio for recurrence or death, 0.68; 95% confidence interval [CI], 0.53 to 0.87; *p* = 0.002 [two-sided]) (See Figure 1). The estimated percentage of patients who remained alive and recurrence-free at 24 months was 77.3% (95% CI, 72.8 to 81.1) in the pembrolizumab group and 68.1% (95% CI, 63.5 to 72.2) in the placebo group. Furthermore, most recently, in a third-interim analysis at 57 months, researchers reported on overall survival benefit with adjuvant pembrolizumab. A statistically significant improvement in OS was observed with pembrolizumab versus placebo (HR 0.62, 95% CI, 0.4420.87; *p* = 0.0024). Pembrolizumab is now Health Canada and FDA Approved for the treatment of clear cell RCC in the adjuvant setting for patients deemed to have intermediate-high to high risk of relapse.

Other studies have also examined the role of immunotherapy in adjuvant settings. Three other major trials have been conducted in this area: CheckMate 914 [17], IMOTION-10 [18], and the PROPSER Trial [19]. As summarized below, these trials had outcomes that differed from those of KEYNOTE-564. For example, in CheckMate-914 [17], 816 patients deemed to be at high risk of recurrence were randomized to receive 6 months of ipilimumab and nivolumab or placebo, and the primary outcome of disease-free survival was not reached (HR for recurrence or death 0.92, 95% CI 0.71–1.19; *p* = 0.53). In IMOTION-10, patients at high risk of recurrence were randomized to receive one year of atezolizumab (PD-L1 Blocker) versus placebo. The primary endpoint of disease-free survival was not statistically different between the two treatment groups. Median disease-free survival (investigator assessment) was 57.2 months (95% CI 44.6 to not estimable) in the atezolizumab group and 49.5 months (47.4 to not estimable) in the placebo group (HR 0.93, 95% CI 0.75–1.15; *p* = 0.50) [18]. In the PROSPER trial, the role of nivolumab was examined in a neoadjuvant setting. Patients received one dose of nivolumab and then underwent nephrectomy. Following this, they received a total of nine doses of nivolumab post-operatively. The trial was stopped prematurely, as there was no difference (HR 0.95) between the two arms of the study. The results of these negative trials clearly conflict with the data from KEYNOTE-564. Potential differences in patient selection, duration of treatment, and agent used (PD-1 versus. PDL-1 inhibitor) could potentially account for the discordant outcomes, and this is currently an active area of investigation [19].

Despite the differences in these trials, based on the results of KEYNOTE-564, Health Canada approved the use of pembrolizumab in the adjuvant setting post-nephrectomy. Patients should have an intermediate or high risk of recurrence, adequate performance status, and no other absolute contraindications to immunotherapy. This approval is for patients specifically with clear cell histology with or without sarcomatoid histology. Furthermore, the current recommendation is to initiate treatment within 12 weeks of surgery [20]. Recently, the Canadian Kidney Cancer Forum released a consensus statement that provides further guidance on the management of RCC in an adjuvant setting [21]. The panel recommended that prior to starting adjuvant treatment, all patients should have baseline CT scans of the thorax, abdomen, and pelvis to exclude early relapse. Patients should undergo imaging every 3–6 months thereafter during their year of pembrolizumab treatment. Following this, patients can be followed up by their urologists, and surveillance can continue as per pre-existing surveillance guidelines based on the patients’ initial risk category [21].

## 3. Selecting First-Line Treatment for Metastatic Clear Cell Renal Cell Carcinoma

In patients with metastatic RCC, the goals of treatment include prolonging survival and improving quality of life, which includes mitigating symptoms of disease and minimizing toxicity. There are several options for managing patients with metastatic RCC in a frontline setting. The number of first-line systemic therapy options for metastatic RCC has increased in recent years, underscoring the importance of appropriate treatment selection. Evidence for active surveillance, single-agent VEGF-targeted therapy, dual-agent immunotherapy, and combination immunotherapy plus VEGF-targeted therapy will be explored, as well as appropriate patient selection and management of treatment toxicity.

### 3.1. Risk-Stratifying Patients with Metastatic RCC 

Risk stratification is an important tool that can inform discussions with patients about prognosis and aid in appropriate treatment selection. The most widely adopted prognostic model in the era of contemporary combination immunotherapy treatment is the International Metastatic RCC Database Consortium (IMDC) risk model. In a seminal study by Heng et al. (2009), 645 patients treated with single-agent sunitinib, sorafenib, or bevacizumab were evaluated using multivariable analysis to determine prognostic factors that were independent predictors of survival [22]. Specific variables that were independently predictive of survival, along with comparative OS data are summarized in Table 1. Next, patients were grouped into three categories: favorable risk (those with 0 of these 6 adverse factors), intermediate risk (those with 1 or 2 of these adverse factors), and poor risk (those with 3–6 of these adverse factors). The 2-year OS rates of these three groups are summarized in Table 2. Subsequently, the IMDC model has been externally validated and widely used in clinical practice to guide treatment decisions [23].

### 3.2. Which Patients Can Be Appropriately Selected for Active Surveillance?

The clinical course of metastatic RCC can vary considerably due to heterogeneity in the underlying tumor biology. Therefore, for some asymptomatic patients with a slow-growing pattern of disease and low disease burden, an active surveillance strategy may be an appropriate strategy. Several studies have supported this strategy [24,25,26]. For example, a prospective study in 2016 enrolled 48 patients with metastatic RCC who were asymptomatic for an active surveillance strategy. Patients underwent CT scans every 3 months for the first year, every 4 months for the second year, and every 6 months thereafter. The average time to the initiation of systemic therapy in this cohort of patients was 14.9 months. IMDC intermediate/poor risk, as well as the number of sites of metastasis, were independent predictors for initiating systemic therapy earlier [24]. A more recent prospective study reported a similar result. Furthermore, quality of life metrics were better in the active surveillance group compared to the treatment group [26]. A study conducted by the Canadian Kidney Cancer Information System compared outcomes in patients who had a delayed start to systemic therapy (defined as starting systemic therapy greater than 6 months after initial diagnosis of metastatic RCC or not started on first-line therapy with an OS greater than 1) versus. Those who were started on systemic therapy within 6 months of diagnosis of metastatic RCC. After adjusting for IMDC risk criteria, they found that an active surveillance strategy was associated with an increased OS and increased time until treatment failure [25]. Based on these studies, an active surveillance strategy may be appropriate for carefully selected patients with appropriate monitoring. Currently, the ASCO Guidelines for the Management of Metastatic RCC support active surveillance in a select population of patients who are asymptomatic, have an IMDC favorable or intermediate risk profile, a favorable histologic profile (low grade and no sarcomatoid features), have a low disease burden, and have had a long interval (greater than 1 year) between initial nephrectomy and diagnosis of metastatic disease [27].

### 3.3. Summary of Evidence for Single-Agent TKI

Advances in the treatment of metastatic RCC have resulted in dramatic improvements in overall survival over the last two decades. Data from the late 1990s indicated that the 5-year OS for metastatic RCC was only between 7 and 10% [28]. The introduction of VEGF-targeted therapies through oral tyrosine kinase inhibitors (TKI) in the management of metastatic RCC was a paradigm shift from previous cytokine-based therapies. VEGF-targeted therapy results in the inhibition of angiogenesis, which is necessary for tumor growth and proliferation. One of the first large trials to show a significant improvement in OS in the era of VEGF-targeted therapy compared the TKI sunitinib with interferon-alpha (INF-alpha) was crucial to the field [29]. In this phase III trial, sunitinib50 mg once daily on 4 weeks ON 2 weeks OFF strategy was compared with INF-alpha three times weekly. The results demonstrated the benefit of sunitinib in terms of the objective response rate (ORR) (47% vs. 12%), progression-free survival (PFS) (11 months for sunitinib vs.. Five months for IFN-alpha), and OS (26.4 vs. 21.8 months, respectively; hazard ratio [HR] 0.821; 95% CI, 0.673 to 1.001; *p* = 0.051) [29]. Pazopanib, another VEGF-targeted multi-kinase inhibitor, was compared to sunitinib in the COMPARZ trial [30]. Patients received either 50 mg of sunitinib on a 4 ON 2 OFF schedule or 800 mg of pazopanib once daily. The results demonstrated that pazopanib was not inferior to sunitinib in terms of PFS and OS. However, the rates of fatigue, myelosuppression, and hand-foot syndrome were lower in patients in the pazopanib group. Patients in the pazopanib group were more likely to have abnormal liver function tests. Taken together, these data demonstrated that pazopanib was similar in efficacy to sunitinib but had more favorable tolerability [30]. In addition to sunitinib and pazopanib, cabozantinib, a potent inhibitor of VEGFR-2, MET, and AXL, has also been studied as a first-line treatment for metastatic RCC. In the CABOSUN trial, cabozantinib was compared to sunitinib in the first-line setting for patients with intermediate and poor-risk disease. The results showed better PFS with cabozantinib (8.2 months; 95% CI, 6.2 to 8.8 months) than with sunitinib (5.6 months; 95% CI, 3.4 to 8.1 months). Furthermore, cabozantinib reduced the rate of disease progression or death by 34% compared with sunitinib (adjusted HR for progression or death, 0.66; 95%, CI 0.46 to 0.95; one-sided *p*=0.012) [31]. These trials have provided strong evidence for the use of TKIs in the first-line treatment of metastatic RCC. However, subsequent trials (as summarized in the next section) focused on combining TKIs and immunotherapy and showed higher efficacy than TKIs monotherapy. Therefore, single-agent TKIs are currently used in the first-line setting for IMDC favorable risk disease and/or where dual therapy would not be tolerated by the patient.

### 3.4. Summary of Evidence for Single-Agent Immunotherapy with TKI

The incorporation of modern immunotherapy agents in the treatment of metastatic RCC has marked another important shift in management. Programmed Death-1 (PD-1) inhibitor nivolumab was demonstrated by Motzer et al. to improve median OS in patients previously treated with one or two lines of VEGF-targeted therapy (25 months; 95% CI 21.8 to not estimable) compared to everolimus (19.6 months; 95% CI 17.6 to 23.1) [32]. Since this pivotal trial, PD-1 inhibitors have been investigated in combination with VEGF-targeted therapies in the first-line setting. KEYNOTE-426, CheckMate 9ER, and CLEAR are phase III clinical trials that have demonstrated an improvement in OS with first-line immunotherapy plus VEGF-targeted combination therapy compared to VEGF-targeted therapy alone [33,34,35]. The results of these trials are summarized in Table 3. KEYNOTE 426 demonstrated that the combination of pembrolizumab and axitinib has an OS improvement, PFS benefit, and better ORR compared to sunitinib. Furthermore, a follow-up to this study demonstrated that these benefits in OS, PFS, and ORR were maintained at 43 months [36]. The CheckMate 9ER trial compared nivolumab and cabozantinib with single-agent sunitinib. Once again, the combination of immunotherapy and TKI provided PFS and OS benefits as well as greater ORR [34]. In addition, this study collected data on quality of life measures using the FKSI-19 and EQ-5 scales and demonstrated that the combination of nivolumab and cabozantinib improved patients’ quality of life compared to sunitinib [37]. These data suggest that despite the higher rate of adverse events in the nivolumab and cabozantinib groups, the overall perceived quality of life was not negatively impacted, which is an important metric in the metastatic setting. 

The CLEAR trial assessed the combination of pembrolizumab and lenvatinib compared to sunitinib in the first-line metastatic setting. In this trial, patients were randomized to one of three groups: pembrolizumab and lenvatinib, lenvatinib and everolimus, or sunitinib. Progression-free survival was longer with pembrolizumab plus lenvatinib than with sunitinib (median, 23.9 versus. 9.2 months; hazard ratio for disease progression or death, 0.39; 95% confidence interval [CI], 0.32 to 0.49; *p* < 0.001). Overall survival was longer with pembrolizumab plus lenvatinib than with sunitinib (hazard ratio for death, 0.66; 95% CI, 0.49 to 0.88; *p* = 0.005) [35]. Taken together, these trials provide strong evidence for the use of a combination of immunotherapy/TKI in the first-line setting for metastatic clear cell RCC. Both the EAU and ASCO guidelines endorse this combination as an acceptable first-line option in IMDC intermediate/poor-risk patients, as well as in IMDC favorable patients. See Table 4 for a full summary of approved combinations in the first-line setting based on the ASCO and EAU Guidelines [27,38].

### 3.5. Summary of Evidence for Dual-Agent Immunotherapy

Combination immunotherapy with nivolumab plus the CTLA4 inhibitor ipilimumab has also demonstrated improved survival compared to VEGF-targeted therapy alone in the first-line setting and has become another standard of care option for IMDC intermediate/poor-risk patients [48]. In the phase III CheckMate-214 trial [48], 1096 patients with clear cell RCC were randomized to receive either four cycles of induction nivolumab/ipilimumab every 3 weeks followed by maintenance nivolumab versus sunitinib for 4 weeks ON and 2 weeks OFF. Notably, patients with PD-L1 expression both above and below 1% were included in the trial. At 18 months, there was an overall survival benefit with nivolumab/ipilimumab compared to sunitinib in the IMDC intermediate/poor-risk disease patients: 75% (95% CI, 70 to 78) versus 60% (95% CI, 55 to 65) (hazard ratio for death, 0.63; 99.8% CI, 0.44 to 0.89; *p* < 0.001), respectively. In addition, there was also a significant difference in the response rate between the two groups favoring nivolumab/ipilimumab, 42% (95% CI, 37–47) versus 27% (95% CI, 22–31), with sunitinib (*p* < 0.001)]. Furthermore, 9% of patients in the nivolumab/ipilimumab had a complete response. In an analysis of favorable risk patients, there was no difference between the two groups in either the overall survival or objective response rate. Although there is a clear benefit in both overall survival and objective response in the dual immunotherapy group, one must consider the functional status of the patient due to the high rates of adverse events. In the CheckMate 214 trial, the rate of grade 3 or 4 events was higher in the sunitinib-only group 63%, compared to 46% for ipilimumab and nivolumab. However, the rate of treatment discontinuation was higher in the ipilimumab and nivolumab groups, 22.7% versus. 13.1%. Moreover, 29.1% of the patients required a course of corticosteroids. Beyond the rate of adverse events, a closer examination of the sub-groups in the study demonstrates that in patients over the age of 75, the hazard ratio for death when comparing ipilimumab and nivolumab to sunitinib was only 0.97 and dual immunotherapy was less beneficial in this sub-population. These findings highlight the importance of appropriate patient selection. More recently, extended 4-year follow-up data for the CheckMate-214 trial have been published [49]. At four years, both OS and PFS benefits continue to be achieved through dual immunotherapy. In the intention-to-treat population, the median OS was not reached at 4 years of follow-up in the ipilimumab and nivolumab group compared to the 38.4 months follow-up with sunitinib (HR 0.69; 95% CI 0.59 to 0.81). In the intermediate to the poor-risk group, the median OS for ipilimumab and nivolumab was 48.1 months versus 26.6 months for sunitinib (HR 0.65; 95% CI 0.54 to 0.78). The 4-year PFS probabilities were 31.0% in the ipilimumab and nivolumab group and 17.3% in the sunitinib group. These data highlight the ongoing benefit of dual immunotherapy after an extended 4-year follow-up period [49].

Beyond dual therapy, a more recent study from 2023, the COSMIC 313 trial, examined the role of combining a dual immunotherapy approach with a TKI [50]. In this study, 855 patients were randomized into one of two groups. The control group received four cycles of q3weekly nivolumab and ipilimumab along with placebo; nivolumab maintenance therapy was continued for up to two years. The experimental group also received dual immunotherapy with ipilimumab and nivolumab, but in addition, they received cabozantinib daily. The results demonstrated improvement with triple therapy compared to dual immunotherapy [probability of progression 0.57 versus. 0.49 (hazard ratio for disease progression or death, 0.73; 95% confidence interval, 0.57 to 0.94; *p* = 0.01).] Overall, the survival data from this study are still pending. Furthermore, there was a significantly greater risk of grade 3 or 4 adverse events in the experimental group than in the control group (79% versus. 56%) [50].

### 3.6. Selection of Optimal Management in the First-Line Setting

The proliferation of treatment options for patients with metastatic RCC has resulted in substantial improvements in survival but has also presented a new challenge of treatment selection from the range of available options.

Active surveillance can be considered for carefully selected, asymptomatic patients with an indolent disease course and a low burden of disease. A long interval from nephrectomy to metastasis, low IMDC prognostic score, and few metastatic sites are favorable features to consider [24,26]. Metastasis-directed therapies, such as surgical resection, radiation, or thermal ablation, should also be considered in selected patients undergoing surveillance with limited disease sites. Careful clinical and radiographic monitoring is essential so that systemic therapy can be implemented at an appropriate time. Immunotherapy combinations with PD-1 inhibitors plus either CTLA-4 inhibitors or VEGF inhibitors have become the preferred first-line options for most patients with metastatic RCC who require systemic therapy. To date, immunotherapy plus VEGF combinations that have demonstrated improved OS compared to VEGF monotherapy include pembrolizumab plus axitinib, pembrolizumab plus lenvatinib, and nivolumab plus cabozantinib [33,34,35]. Avelumab (PD-L1 inhibitor) plus axitinib has demonstrated an improvement in PFS, but not OS, over VEGF monotherapy (JAVELIN 101) and is approved as first-line therapy for metastatic RCC [51]. Currently, ipilimumab plus nivolumab is approved for use in IMDC intermediate- and poor-risk patients only and not favorable-risk patients [49].

No studies have directly compared immunotherapy combinations in a head-to-head manner. A retrospective cohort analysis of real-world data has provided prognostic benchmarks for patients with metastatic RCC receiving contemporary first-line systemic therapy stratified by the IMDC risk group. Patients with non-clear cell RCC were also included in this study. OS at 18 months for favorable, intermediate, and poor-risk groups were 90%, 78%, and 50% for those receiving ipilimumab and nivolumab, 93%, 83%, and 74% for immunotherapy plus VEGF-targeted therapy, and 84%, 64%, and 28% for those receiving VEGF monotherapy, respectively. The overall response rate for favorable, intermediate, and poor-risk was 41.3%, 40.6%, and 33% for dual immunotherapy; 60.3%, 56.8, and 40.9% for immunotherapy plus VEGF-targeted therapy; and 39.3%, 33.5%, and 20.9% for VEGF-targeted therapy, respectively [52].

For patients with IMDC favorable risk metastatic RCC, immunotherapy plus VEGF-targeted therapy combinations are the preferred first-line systemic therapy for most patients. Although an OS advantage has not been demonstrated in IMDC favorable risk in subgroup analysis, immunotherapy plus VEGF combinations remain the preferred choice due to better ORR and PFS [35,53]. Single-agent VEGF-targeted therapy with pazopanib or sunitinib may still be appropriate for patients who are unfit for combination therapy, have comorbidities that preclude immunotherapy, or are based on patient goals of care. In patients for whom VEGF monotherapy is chosen, single-agent immunotherapy may be considered in the second-line setting if appropriate.

For patients with IMDC intermediate- or poor-risk metastatic RCC, the preferred first-line treatment for most patients will include a PD-1 inhibitor backbone combined with either VEGF-targeted therapy or CTLA-4 inhibitor. Ipilimumab plus nivolumab is a standard option for patients with the goal of long-term, durable responses and possible treatment-free intervals. However, in CheckMate 214, progressive disease was the best response for intermediate- and poor-risk patients at 19.3% [49]. Therefore, close observation is necessary to identify those patients with primary progressive disease who may benefit from changing to second-line VEGF-targeted therapy to prevent clinical decline. In addition, pembrolizumab plus axitinib, pembrolizumab plus lenvatinib, and nivolumab plus cabozantinib remain standard care options for patients with intermediate/poor-risk disease. In contrast to the relatively high rates of primary progression with ipilimumab and nivolumab, the rates of primary progressive disease with immunotherapy plus VEGF-targeted therapy were 10.9% or less [35,53,54]. Therefore, for patients with rapidly progressing or a high burden of disease in whom a rapid response is required, immunotherapy plus VEGF-targeted therapy should be considered over dual immunotherapy. In patients with intermediate/poor-risk disease who have comorbidities that limit the use of immunotherapy, such as significant autoimmune disease, a solid organ transplant, required immunosuppressive medications, or poor performance status, treatment with VEGF-targeted therapy in the first-line setting may be indicated. The phase II CABOSUN trial highlighted improved PFS and ORR with cabozantinib compared to sunitinib in the first-line setting; before, the choice of first-line VEGF-targeted therapies included cabozantinib, pazopanib, and sunitinib [55].

Tumor histology may also influence optimal treatment selection. RCC with sarcomatoid features has been recognized as an independent predictor of poor prognosis [56]. In the era of immunotherapy, however, patients with sarcomatoid features may derive greater benefits from immune checkpoint inhibitor therapies and fewer benefits from VEGF-targeted therapies [57]. Therefore, combination immunotherapy with ipilimumab and nivolumab may be favored over immunotherapy plus VEGF-targeted therapy and immunotherapy plus VEGF-targeted therapy over VEGF-targeted monotherapy when sarcomatoid features are present.

To date, there is limited evidence to guide first-line treatment selection in patients with metastatic RCC who have received adjuvant immunotherapy. For patients who develop recurrence within a short interval from adjuvant immunotherapy, VEGF-targeted therapy would be preferred; however, this area remains an unmet need for future research.

Ultimately, the selection of optimal first-line systemic therapy must take into consideration available evidence, local availability of treatment options, patient fitness and comorbidities, exposure to adjuvant immunotherapy, and patient goals and wishes. A shared decision model with patient discussions is recommended during treatment selection.

### 3.7. Addressing the Role of Cytoreductive Nephrectomy

Cytoreductive nephrectomy (CN) was established as the standard of care for patients with mRCC in 2011 in the cytokine era prior to the availability of targeted therapies and immunotherapy. A combined analysis of two prospective randomized trials using identical protocols comparing cytoreductive nephrectomy plus interferon-alpha-2b versus interferon-alpha-2b alone in patients with metastatic renal cancer and good performance status ECOG 0 or 1 resulted in a median survival of 13.6 months for nephrectomy plus interferon versus 7.8 months for interferon alone [58]. A retrospective analysis by Anderson suggested that patients most likely to benefit from CN before systemic therapy were those with lung-only metastases, good prognostic features, and a good performance status [59].

With the advent of more effective systemic therapies, the role of CN has become less clear. A large retrospective analysis by Heng et al. of 1658 patients with synchronous mRCC showed that the HR for death for patients undergoing CN was 0.60 (95% confidence interval, 0.52–0.69; *p* < 0.0001) but that patients with four or more IMDC risk factors did not benefit from CN [60]. Another large retrospective multi-institutional analysis [61] of over 1900 patients suggested a 12-month improvement in median OS for patients undergoing CN (26.6 versus 14.6 months, *p* < 0.001). CARMENA is the only prospective randomized trial to address the role of CN in the targeted therapy era, and patients were randomized to CN followed by sunitinib compared to sunitinib monotherapy. Sunitinib alone was found to be non-inferior to sunitinib plus CN in terms of OS, suggesting that immediate CN does not benefit all patients [62]. A post hoc analysis reported that for patients with only one IMDC risk factor, OS was longer after nephrectomy (31.4 versus 25.2 months) whereas for patients with two or more IMDC risk factors, OS was better for patients who did not undergo CN (31.2 months versus 17.6 months, respectively; HR, 0.65; *p* = 0.03), emphasizing the importance of patient selection when considering CN [63].

The SURTIME trial compared the strategy of upfront CN followed by sunitinib versus delayed CN for patients with no evidence of progression after 3 months of sunitinib [64]. The intention-to-treat OS hazard ratio of deferred versus immediate CN was 0.57 (95% CI, 0.34–0.95; *p* = 0.03), with a median OS of 32.4 months (95% CI, 14.5–65.3 months) in the deferred CN arm and 15.0 months (95% CI, 9.3–29.5 months) in the immediate CN arm, suggesting that for patients with intermediate or poor-risk disease treated with targeted therapy, CN should only be offered if there is evidence of benefit from initial systemic therapy.

Large retrospective observational studies have reached different conclusions. A study by the International Metastatic Renal Cell Carcinoma Database [65] examined the effect of upfront CN on OS in patients with de novo mRCC who received targeted therapy or immunotherapy. Among the 4202 patients treated with targeted therapy, 2326 (55%) underwent upfront CN, as did 234 of the 437 patients (54%) treated with checkpoint inhibitors. CN was associated with an HR for death of 0.72 (95% CI, 0.67–0.78, *p* < 0.001) for the targeted therapy group and 0.61 (95% CI, 0.41–0.90, *p* = 0.013) for the immunotherapy group, suggesting that upfront CN is associated with OS benefit in select patients even in the age of more effective systemic therapies.

However, a cohort study using data derived from the National Cancer Database reached a different conclusion. From 1 January 2006 to 31 December 2016, 12,766 patients with metastatic clear cell RCC who received targeted therapy were identified, of whom 5005 (39%) underwent CN. The authors used instrumental variable analysis to adjust for unmeasured confounding factors in the estimation of the effect of CN on OS. Instrumental variable analysis failed to show an association between CN and OS (HR 0.92, 95% CI, 0.78–1.09) [66].

To better select patients who might benefit from CN, Marchioni et al. used data from the Registry for Metastatic RCC (REMARCC) to develop a scoring system to predict the overall mortality (OM) in patients with mRCC undergoing upfront CN [67]. A total of 519 patients with synchronous mRCC who underwent CN between 2005 and 2019 were identified. OM was highest in patients with bone, liver, or lung metastases, those with a performance status (PS) <80%, and those with more than three metastases, and was lower in obese patients. This study suggests that patient factors (PS, obesity) and tumor factors (location and number of metastases) should be taken into consideration along with IMDC and MSKCC risk scores when selecting patients for upfront CN.

Uncertainty exists regarding the optimal timing of the CN. Meahger et al. performed a retrospective analysis using data from REMARCC and found that better OS was observed in patients who received systemic therapy prior to undergoing CN (HR 0.67, *p* = 0.039) [68]. This result is in keeping with the findings of SURTIME which found a trend toward improved OS in the deferred vs. the upfront CN arm (HR 0.57 (95% CI, 0.34–0.95; *p* = 0.03)) with a median OS of 32.4 months (95% CI, 14.5–65.3 months) in the deferred CN arm vs. 15.0 months (95% CI, 9.3–29.5 months) in the immediate CN arm in the ITT population although this difference was no longer statistically significant in the per-protocol population (HR, 0.71; 95% CI, 0.40–1.24, *p* = 0.23). Overall, these findings suggest that deferring CN until after the initiation of systemic therapy might allow for a better selection of patients who might benefit from surgery.

Trials assessing the potential benefits of deferred CN in the era of immunotherapy and combination therapy are ongoing. NORDIC-SUN and PROBE [69] are both randomized phase III trials comparing the effect of deferred CN with no CN following initial therapy with an IO/IO or TKI/IO combination in mRCC patients with a primary endpoint of OS.

While awaiting these results, the available data suggest that the role of CN should be individualized, considering factors such as patient performance status, tumor burden, and response to initial systemic therapy. CN should not be routinely recommended for patients with poor performance status or a poor risk of disease.

### 3.8. Addressing Intracranial Disease with Systemic Therapy with or Without Local Therapy

The incidence of brain metastases associated with metastatic renal cell carcinoma is approximately 10% [70]. Although survival has improved over the past several years, the median survival among patients with intracranial disease ranges between 4 and 35 months [67]. Graded prognostic estimates are readily available for prognostication and guide management [71].

#### 3.8.1. Studies Evaluating Immunotherapy

In patients with treatment-naïve CNS metastases, neurosurgery and/or radiosurgery are the preferred modalities to optimize disease control prior to initiation of frontline systemic therapy, given their risk of intracranial hemorrhage. The optimal choice of systemic therapy for this cohort is rapidly evolving [72,73,74,75,76,77].

In the nonrandomized, open-label phase 2 trial GETUG-AFU 26 NIVOREN, 73 patients with metastatic RCC and asymptomatic CNS metastases received single-agent nivolumab in the second-line setting after progression to an angiogenesis inhibitor [75]. Half of the participants did not receive prior brain-directed therapy, whereas the other half previously received either stereotactic radiosurgery or whole-brain radiation. After a median follow-up of 2 years, among the 39 patients with untreated brain metastases, the intracranial objective response rate was 12%, and median intracranial progression-free survival was 2.7 months. On the other hand, there were no objective response rates in the 39 patients with prior brain radiotherapy. Grade 3 toxicities were reported in 12% of the participants, and no treatment-related grade 5 events occurred.

In the nonrandomized open-label phase II trial CheckMate 920 [76], 28 patients with asymptomatic CNS metastases that were treatment-naïve RCC received combination therapy with ipilimumab and nivolumab according to the standard protocol. Responses were observed in 32% of the participants, whereas the median progression-free survival was 9 months. Relevant grade ≥3 central nervous system toxicities related to immunotherapy exposure included diarrhea, colitis, diabetic ketoacidosis, hepatitis, hypophysitis, and skin rash.

#### 3.8.2. Studies Evaluating Antiangiogenic Agents

Studies evaluating angiogenesis inhibitors have shown some intracranial efficacy; however, the durability of responses and safety in this setting are unconfirmed. Furthermore, these agents may be associated with an increased risk of intracerebral hemorrhage [77].

In an open-label, expanded international access program study, investigators assessed the efficacy and safety of sunitinib at 50 mg daily for 4 out of 6 weeks in patients with previously treated and treatment-naïve metastatic renal cell carcinoma. In 4564 patients, baseline brain metastases were observed in 321 (7%) participants. The meeting treatment duration was three cycles (range, 1–25). Of 213 evaluable patients, 26 (12%) had an objective response (progression-free survival and overall survival were 5.6 months (95% CI, 5.2–6.1) and 9.2 months (95% CI, 7.8–10.9), respectively). The most common grade 3 toxicities included fatigue and ischemia (7% each), thrombocytopenia (6%), and neutropenia (5%) [73].

A retrospective study evaluated the incidence of brain metastases in patients with metastatic RCC who were randomized to sorafenib 400 mg twice daily in the phase III Treatment Approaches in the Renal Cancer Global Evaluation Trial (TARGET) [78]. The overall incidence of brain metastases in participants receiving sorafenib was 3% compared with 12% in patients receiving placebo. Similarly, the incidence of brain metastases was significantly lower in the sorafenib group than in the placebo group at 1 (*p* = 0.0447) and 2 years (*p* = 0.005). The authors concluded that sorafenib may reduce the recurrence of metastases in this cohort and could be an effective preventive therapy for CNS metastases associated with RCC.

The clinical activity and safety profile of cabozantinib monotherapy in any line of treatment were assessed in patients with brain metastases from metastatic RCC participating in an international retrospective double-cohort study. In cohort A, 38% (33/88) of patients had progressing brain metastases without brain-directed local therapy, whereas 62% (55/88) of patients in cohort B had stable or progressing brain metastases and received brain-directed local therapy. At a median follow-up of 17 months, the intracranial response was 55% (95% CI, 9.0–30.0 months) and 47% (95% CI, 33–61%) in cohorts A and B, respectively. In cohort A, the median time to treatment failure was 8.9 months (95% CI, 5.9–12.3 months), and the median overall survival was 15 months (95% CI, 9.0–30.0 months). In cohort B, time to treatment failure was 9.7 months (95% CI, 6.0–13.2 months), and median overall survival was 16 months (95% CI, 12.0–21.9 months). Cabozantinib exposure was not associated with any grade ≥3 toxic effects [79].

## 4. Selection of Second-Line Treatment for Metastatic Clear Cell Renal Cell Carcinoma

The selection of second-line therapy is generally influenced by prior drug exposure and toxicity profile, either in the first-line or adjuvant setting. Therefore, participation in clinical trials is highly recommended.

Cabozantinib is an oral inhibitor of tyrosine kinases, including MET, VEGFR, and AXL. MET and AXL have been implicated in tumor resistance in patients previously treated with VEGFR tyrosine kinase inhibitor monotherapy [79,80].

METEOR was a randomized phase 3 study that compared cabozantinib against the mTOR inhibitor everolimus in patients with progressive disease after either one of sunitinib, pazopanib, axitinib, sorafenib, bevacizumab, interleukin-2 or interferon-alpha or nivolumab. Nivolumab was given to 5% of patients for both arms. Of note, thirty percent of patients had received two or more lines of therapy before enrollment. In this trial, 658 patients were randomized, 330 received cabozantinib, and 328 received everolimus. Median overall survival was 21.4 months (95% CI 18.7—not estimable) with cabozantinib and 16.5 months (14.7–18.8) with everolimus (HR 0.66 [95% CI 0.53–0.83]; *p* = 0.00026). Cabozantinib also improved progression-free survival (HR 0·51 [95% CI 0·41–0·62]; *p* < 0·0001) whereas objective response rate also favored cabozantinib over everolimus (17% (3–22) versus 3% (2–6); *p* < 0.0001, respectively). Grade ≥3 adverse events were more frequently observed with cabozantinib when compared to everolimus and included hypertension (15% versus 4%), diarrhea (13% versus 2%), fatigue (11% versus 7%), and palmar-plantar erythrodysesthesia syndrome (8% versus 1%). Careful monitoring for adverse events is recommended, and dose modifications should be considered for patients with grades 2–3 toxic effects [39].

AXIS was a phase 3 clinical trial that enrolled 723 patients with confirmed clear cell renal cell carcinoma who had progressed after first-line therapy with either sunitinib, bevacizumab plus interferon-alpha, temsirolimus, or cytokines and randomized them in a 1-1 fashion to either axitinib or sorafenib. The axitinib dose was 5 mg p.o. twice daily; it could be increased to 7 mg and then to 10 mg twice daily for patients who did not experience hypertension or any adverse reaction higher than grade 2. The primary endpoint was progression-free survival. In the final analysis, median progression-free survival was 8.3 months with axitinib versus 5.7 months with sorafenib, for a one-sided *p* less than 0.0001. Median overall survival was 20.1 months for axitinib versus 19.2 months for sorafenib, with one-sided *p* = 0.3744. The median duration of response was 11 months for axitinib and 10.6 months for sorafenib [40,41].

## 5. Patients with Progressive Disease Despite Multiple Prior Lines of Therapy

RECORD1 was a phase III trial that enrolled 410 patients with metastatic renal cell carcinoma that had a clear cell component who had received one or prior lines of therapy and randomized them 2-1 to either everolimus 10 mg p.o. daily or placebo until disease progression, intolerable toxicity, or death. Previous therapies included, for the everolimus and the placebo groups, respectively: Sunitinib 46/43%, sorafenib 28/30%, both sunitinib and sorafenib 26/26%, interferon 51/52%, interleukin-2 22/24%, chemotherapy 13/16%, bevacizumab 9/10%. Nephrectomy occurred in 96/95% of cases, respectively. The primary endpoint was progression-free survival, the median was four versus 1.9 months, and the trial had to be halted at the second interim analysis due to a significant difference in efficacy. The partial response rate is 1.8% with everolimus, 0% with placebo, stable disease 66.8% with everolimus, and 32.4% with placebo [42]. 

CheckMate 025 was a phase 3 study that enrolled 821 patients with advanced renal cell carcinoma that had a clear cell component who had been previously treated with one or two regimens of antiangiogenic therapy and randomized them in a one-to-one ratio to receive either nivolumab or everolimus. The primary endpoint was overall survival. The median overall survival was 25 months (95% confidence interval 21.8 to not measurable) with nivolumab and 19.6 months (95% confidence interval 17.6–23.1) with everolimus. The hazard ratio for death is 0.73, and P equals 0.002, meeting the previously specified threshold for superiority. Median progression-free survival was 4.6 months for nivolumab and 4.4 months for everolimus, with *p* = 0.11. Previous systemic therapies included sunitinib, pazopanib, or axitinib. 72% of the patients enrolled had only received one line of therapy, whereas 28% had been exposed to two lines [32].

An open-label multicenter phase II clinical trial compared lenvatinib 24 mg p.o. daily, everolimus 10 mg p.o. daily, and the combination (lenvatinib 18 mg p.o. daily and everolimus 5 mg p.o. daily) in a 1:1:1 ratio in patients with histologically verified clear cell renal cell carcinoma and evidence of disease progression within 9 months of stopping previous treatment. Prior treatments included axitinib, bevacizumab, pazopanib, sorafenib, sunitinib, and cabozantinib. Sunitinib represented 56% of all prior treatments, and pazopanib 26%. The third most common agent was bevacizumab at 8%. The primary endpoint was progression-free survival. The combination was significantly better than single-agent lenvatinib, with a hazard ratio 0.40, 95% CI 0.24–0.68, *p* = 0.0005. In turn, lenvatinib was superior to everolimus, with a hazard ratio of 0.61, 95% confidence interval of 0.38–0.98, *p* = 0.048. The median progression-free survival was 14.6 months for the combination (95% CI --5.9–20.1), 7.4 months for single-agent lenvatinib (95% CI 5.6–10.2), and 5.5 months for single-agent everolimus (95% CI 3.5–7.1). Median overall survival was 25.5 months for the combination (95% CI 16.4-not reached), 19.1 months for single-agent lenvatinib (95% CI 13.6–26.2), and 15.4 months for single-agent everolimus (95% CI 11.8–19.6) [43].

CONTACT-03 was a phase III clinical trial that enrolled 522 patients with locally advanced or metastatic renal cell carcinoma that had progressed on an immune check point inhibitor and randomized them to atezolizumab plus cabozantinib or cabozantinib monotherapy. The study failed to show the superiority of the former, with a non-significant median progression-free survival of 10.6 versus 10.8 months, respectively, with a 95% confidence interval for the monotherapy group of 9.8–12.3 months. Cabozantinib monotherapy had an objective response rate of 41% by central review (95% CI 35–47%) in this patient population. The first line of therapy in this group included ipilimumab-nivolumab in 27%, sunitinib in 28%, pazopanib in 17%, and axitinib-pembrolizumab in 11%. Second-line nivolumab had been used in 93% of cases, and 37% of patients had not been exposed to a previous VEGF tyrosine kinase inhibitor [44].

Telaglenastat is a reversible inhibitor of glutaminase, a key enzyme in the conversion of glutamine to glutamate and alpha-ketoglutarate. This process is key to the supply of carbon molecules through the Warburg effect of increased glycolysis and glutamine utilization by cancer cells. Based on phase I data, telaglenastat is being explored in phase II and later-line trials in patients with renal cell carcinoma.

CANTATA was a phase II randomized clinical trial of cabozantinib with telaglenastat or placebo. The study enrolled 444 patients and randomized them in a 2:1 ratio. Prior 1, 2, or 3 lines of therapy relationships for each of the telaglenastat–cabozantinib versus placebo–cabozantinib arms were 57/57% for first-line, 43/42%, and 0/1%, respectively. In both arms, 62% of patients had received prior ICI therapy, of which 29% received ipilimumab plus nivolumab. The objective response rate was 31% for telaglenastat and cabozantinib, and 28% for placebo plus cabozantinib. Median progression-free survival, the main outcome, was 9.2 months for telaglenastat plus cabozantinib versus 9.3 months for placebo–cabozantinib, and this difference was significant [45].

ENTRATA was a phase II randomized clinical trial that evaluated everolimus with telaglenastat or placebo. Partial response with telaglenistat–everolimus was 2.2% and 0% in the placebo-everolimus arm. Stable disease was 56.5% for telaglenastat–everolimus and 47.8% with the placebo-everolimus arm. Median progression-free survival, the primary endpoint, was 3.8 months for the telaglenastat–everolimus arm versus 1.9 months for the placebo-everolimus arm. These differences were considered significant [46].

Under normoxic conditions, hypoxia-inducible factor alpha (HIF-α) undergoes hydroxylation and ubiquitylation for subsequent degradation in the proteasome. Under hypoxic conditions, however, this process does not occur, and HIF-α concentrations increase intracellularly, subsequently entering the nucleus, thereby inducing target genes, including erythrophyetin (EPO), VEGF, HO-1, ADM, and Glut-1, which then suppress apoptosis and promote cell survival. HIF2-alpha (HIF2-α) is a novel target in the treatment of renal cell carcinoma, as it plays a role in the metabolism, survival, proliferation, angiogenesis, and metastasis of renal cell carcinoma. Belzutifan is a direct HIF2a inhibitor [81].

LITESPARK-005 was a phase III clinical trial that enrolled 374 patients previously treated with up to three lines of therapy, which should have included an anti-PD-(L)1 and a VEGF tyrosine kinase inhibitor, randomized them 1:1 to belzutifan or everolimus. The dual primary endpoints were progression-free survival and overall survival. Median progression-free survival was 5.6 months for belzutifan versus 5.6 months for everolimus, with a hazard ratio of 0.74 (95% CI 0.63–0.88), with significant *p*-value < 0.001 from the first interim analysis. Median overall survival was 21.4 months for belzutifan versus 18.1 months for everolimus, with a hazard ratio of death 0.88 (95% CI 0.73–1.07), with *p* = 0.09941 at the second interim analysis. The objective response rate was 22.7% for belzutifan (95% CI 18.6–27.3) and 3.5% (95% CI 1.9–5.9) for everolimus. This data led to FDA approval of belzutifan for refractory clear cell renal cell carcinoma [47].

## 6. Future Directions

### 6.1. Ongoing Trials

Important studies are currently underway to assess the efficacy of intensified adjuvant regimens in patients with disease at intermediate-high or high risk of distant relapse, while others assess outcomes for patients having experienced disease progression in the advanced setting. A few selected examples are highlighted below.

LITESPARK-022 (NCT05239728) is aimed at addressing an area of unmet need, namely, patients at intermediate-high or high risk of disease relapse despite adjuvant pembrolizumab (see Section 2 above). Belzutifan is a novel hypoxia-inducible factor-2α inhibitor (MK-6482) that has shown activity and favorable tolerability in patients with advanced clear cell RCC and von Hippel Lindau (VHL) disease-associated RCC [47]. In this global, multicenter, double-blind, randomized, phase III study, approximately 1600 participants with intermediate-high [pT2, grade 4 or sarcomatoid, N0, M0 or pT3 any grade, N0, M0], high [pT4, any grade, N0, M0 or pT, any stage/grade, N+, M0], or M1 NED) will be randomized to receive belzutifan 120 mg orally once daily and pembrolizumab 400 mg intravenously (IV) every 6 weeks (Q6W) or oral placebo and pembrolizumab 400 mg IV Q6W for one year. The primary endpoint for the study is disease-free survival, whereas the key secondary endpoint is overall survival. Additional secondary endpoints include safety, disease recurrence = specific survival, and patient-reported outcomes [82].

Next, the LITESPARK-011 study is an open-label, multicenter, randomized, phase III study designed to compare the efficacy and safety of belzutifan and lenvatinib versus cabozantinib monotherapy in participants with advanced ccRCC following progression on or after anti-PD-1/L1 therapy in the first- or second-line. The co-primary endpoints include PFS and OS, whereas the secondary endpoints include ORR, duration of response (DOR), and safety/tolerability. Approximately 700 participants will be recruited globally. Study treatment will be continued until documented progression, initiation of a new anticancer treatment, unacceptable toxicity, or patient withdrawal [83].

### 6.2. Theranostics

Radionuclide-based therapy represents a promising new class of antineoplastic therapy whereby biomolecules (e.g., monoclonal antibodies) are radiolabeled and target a specific tumor antigen or receptor, allowing for concentration of the drug within the tumor microenvironment [84].

^177^Lutetium is a heavy metal that can emit β-rays over a very short distance (760 μm), thereby limiting damage to nearby healthy tissues [85].

Prior studies have suggested that carbonic anhydrase IX is nearly ubiquitously expressed (>90%) in metastatic ccRCC; it is also present in gastrointestinal mucosal cells and associated structures. Radiolabeling of the anti-CAIX monoclonal antibody girentiuxmab with 177Lu has shown promising results in ccRCC [86].

The phase II STARLITE 2, open-label, single-arm study will attempt to build on these results by combining nivolumab with the ^177^Lu-labeled anti-carbonic anhydrase IX (CAIX) monoclonal antibody girentuximab (^177^Lu-girentuximab) in patients with previously treated advanced ccRCC. Participants will receive ^177^Lu-girentuximab every 12–14 weeks for a maximum of 3 doses plus nivolumab 240 mg every 2 weeks until disease progression or unacceptable toxicity. The primary endpoint will be the response rate within 24 weeks, whereas secondary endpoints will include PFS, OS, and toxicity [87].

## 7. Putting it All Together: Practical Recommendations for General Practitioners in Oncology Managing RCC

### 7.1. Review of Toxicities for TKI’s and Immunotherapy

The main toxicities associated with TKIs include diarrhea, stomatitis, hypertension, hand-foot syndrome, and fatigue [88]. All patients who start TKI therapy should be asked to monitor their home blood pressure. Specific lab abnormalities that the clinician should be aware of include increased creatinine (highest incidence of this with Lenvatinib), hyperglycemia or less commonly hypoglycemia (more frequently with Lenvatinib and Axitinib), increased LFT, and electrolyte abnormalities (hyponatremia, hypocalcemia, hypomagnesemia) [84]. Furthermore, blood pressure should be checked routinely as part of patients’ follow-up visits. Diarrhea can be managed using Imodium, as per previously published CCO guidelines [89]. However, other causes of diarrhea should also be investigated and treated as necessary. Less common but serious side effects associated with TKIs include the risk of gastrointestinal perforation, complications with wound healing, venous and arterial thromboembolism, severe hemorrhage, and cases of arterial dissection. Patients should be counseled to inform their cancer care team prior to any surgical procedure. In general, TKIs should be administered 28 days before surgery. Immunotherapy can cause a broad range of immune-related adverse events. These include, but are not limited to, skin/cutaneous toxicity, hypothyroidism, adrenal insufficiency, colitis, pneumonitis, myocarditis, immune-related diabetes, nephritis, hepatitis, pancreatitis, myasthenia gravis, and arthritis. For full guidelines on the management of these toxicities please refer to the Cancer Care Ontario Immune Checkpoint Inhibitor Side Effect Toolkit (Immune Checkpoint Inhibitor Side Effect Toolkit|Cancer Care Ontario, https://www.cancercareontario.ca/en/guidelines-advice/modality/immunotherapy/immune-therapy-toolkit, accessed on 15 July 2024) [90] and ESMO Guidelines referenced here [91].

### 7.2. What Regimen is the Most Appropriate First-Line Treatment for My Patient?

Table 5 and Table 6 summarize key recommendations for first-line therapy in metastatic clear cell RCC for favorable as well as intermediate and poor-risk category patients, respectively. Figure 1 provides an algorithm to help guide decision-making when selecting treatment for a given patient.

For favorable-risk patients, guidelines recommend either TKI combined with immunotherapy or a single-agent TKI. The data presented above summarize the observed benefits of progression-free survival, overall survival, and response rates favoring combination therapy. Reasons to treat a patient with a single-agent TKI may include an absolute contraindication to immunotherapy, poor patient performance status, or patient preference. Between single-agent sunitinib and pazopanib, the COMPARZ trial generally found that pazopanib was better tolerated by patients than sunitinib [30]. However, compared to sunitinib, pazopanib does carry a higher risk of drug-induced liver injury. For IMDC intermediate- and poor-risk diseases, five approved combinations are summarized below. To date, there are no prospective trials comparing these regimens; therefore, ASCO recommends that therapy be selected based on patient preference and predicted tolerability [27].

## 8. Conclusions

Promising novel therapeutic strategies for patients with renal cell carcinoma have led to improved outcomes with acceptable toxicities. Modern risk stratification instruments can inform the management of metastatic disease in first- and later-line settings. Local strategies may be appropriate in select cases with support from multidisciplinary case conferences. General practitioners in oncology play a vital role in certain geographic areas, such as Canada, vis-à-vis the co-management of patients affected by renal cell carcinoma. Collaborative efforts with medical oncologists, such as this article, are highly encouraged to continuously develop knowledge and experience to optimize care for patients with renal cell carcinoma. International recommendations from the Canadian Urologic Association, Canadian Kidney Cancer Forum, European Society of Medical Oncology, and American Urologic Association are excellent resources and readily available online.

## Figures and Tables

**Table 1:** Summary of the IMDC prognostic model with comparative overall survival data (This data is presented for relative comparison only, as overall survival data pre-dates the era of Immunotherapy and Targeted Agents). Data from [22].

IMDC Prognostic Model Factors which Independently Predict Overall Survival *
Hemoglobin less than the lower limit of normal (*39.4 months versus. 16.9 months*)
Karnofsky Performance Status less than 80% (*31.6 months versus. 9.4 months*)
Serum Corrected Calcium Above the Upper Limit of Normal. (*26.8 months versus. 8.8 months*)
Neutrophils greater than the Upper Limit of Normal (*27.0 months versus. 5.9 months*)
Platelets Greater than the Upper Limit of Normal (*27.4 months versus. 10.4 months*)
Less than 1 year of time between initial diagnosis and systemic therapy (*30.9 months versus.15.8 months*)

* Survival rates are based on data from the pre-immunotherapy era.

**Table 2:** Summary of 2-year overall survival data for the IMDC risk groups.

Risk Category	2-Year OS *
Favorable Risk	75%
Intermediate Risk	53%
Poor Risk	7%

* Survival rates are based on data from the pre-immunotherapy era.

**Table 3:** Summary of clinical trials assessing immunotargeted agents in combination for first-line metastatic RCC.

Trial Name	Immunotherapy/TKI Combo	PFS Data	OS Data	Objective Response Rate
KEYNOTE-426 [33]	Pembrolizumab/Axitinib versus sunitinib [12.8 months follow-up].	15.1 months versus 11.1 months (hazard ratio for disease progression or death, 0.69; 95% CI, 0.57 to 0.84; *p* < 0.001)	89.9% versus 78.3% (hazard ratio for death, 0.53; 95% confidence interval [CI], 0.38 to 0.74; *p* < 0.0001)	59.3% (95% CI, 54.5 to 63.9) versus 35.7% (95% CI, 31.1 to 40.4) in the sunitinib group (*p* < 0.001)
CheckMate 9ER [34]	Nivolumab/Cabozitinib versus sunitinib [18.1 months follow-up]	The median progression-free survival was 16.6 months (95% confidence interval [CI], 12.5 to 24.9) with nivolumab plus cabozantinib and 8.3 months (95% CI, 7.0 to 9.7)	The probability of overall survival at 12 months was 85.7% (95% CI, 81.3 to 89.1) with nivolumab plus cabozantinib and 75.6% (95% CI, 70.5 to 80.0) with sunitinib (hazard ratio for death, 0.60; 98.89% CI, 0.40 to 0.89; *p* = 0.001).	Objective response occurred in 55.7% of the patients receiving nivolumab plus cabozantinib and in 27.1% of those receiving sunitinib (*p* < 0.001)
CLEAR [35]	Pembrolizumab/Lenvatinib versus sunitinib	Median 23.9 versus 9.2 months; (hazard ratio for disease progression or death, 0.39; 95% confidence interval [CI], 0.32 to 0.49; *p* < 0.001).	(At 24-month follow-up) 79.2% versus 70.4% hazard ratio for death, (0.66; 95% CI, 0.49 to 0.88; *p* = 0.005)	71.0% versus 36.1% relative risk with lenvatinib plus pembrolizumab versus. sunitinib, 1.97 [95% CI, 1.69 to 2.29]

**Table 4:** Summary of clinical trials assessing second- or later-line therapy.

Trial	Phase	Prior Lines of Therapy	Prior ICI in >50% of Patients	Comparison	Main Outcome
METEOR [39]	3	Multiple lines of therapy were allowed so that 70% of patients had only received one line, and 30% had received two or more lines. These included either one of sunitinib, pazopanib, axitinib, sorafenib, bevacizumab, interleukin-2, or interferon-alpha or nivolumab.	Nivolumab was not given to 5% of patients for both arms.	Cabozantinib vs. everolimus	mOS 21.4 mo with cabozantinib vs. 16.5 mo with everolimus, significant
AXIS [40,41]	3	Sunitinib, bevacizumab plus interferon-alpha, tensirolimus, or cytokine.	No	Axitinib vs. sorafenib	mPFS 8.3 mo with axitinib vs. 5.7 mo with sorafenib, significant, overall survival also significant
RECORD1 [42]	3	Sunitinib 46/43%, sorafenib 28/30%, both sunitinib and sorafenib 26/26%, interferon 51/52%, interleukin-2 22/24%, chemotherapy 13/16%, bevacizumab 9/10%.	No	Everolimus vs. placebo	mPFS 4 mo with everolimus vs. 1.9 mo with placebo, significant
CheckMate 025 [32]	3	One line of therapy in 72%, and the rest received up to 2. These included either one of sunitinib, pazopanib or axitinib.	No	Nivolumab vs. everolimus	mOS 25 mo with nivolumab vs. 19.6 mo with everolimus. Significant
Lenvatinib vs. Everolimus vs. both [43]	2	Axitinib, bevacizumab, pazopanib, sorafenib, sunitinib, antibozitinib, and all VEGF inhibitors	No	Lenvatinib vs. everolimus vs. both	mPFS 14.6 mo for lenvatinib-everolimus, 7.4 mo for lenvatinib monotherapy, and 5.5 mo for everolimus monotherapy. The combination was significantly better than lenvatinib monotherapy. mOS 25.5 mo with combination vs. 19.1 mo with lenvatinib monotherapy
CONTACT-03 [44]	3	The first-line therapy in this group included ipilimumab-nivolumab in 27%, sunitinib in 28%, pazopanib in 17%, and axitinib-pembrolizumab in 11%. Second-line nivolumab had been used in 93% of cases, and 37% of patients had not been exposed to a previous VEGF tyrosine kynase inhibitor.	Yes	Atezolizumab-cabozantinib (ICI rechallenge) vs. cabozantinib monotherapy	PFS and OS not better with atezolizumab rechallenge. Note: ORR was 41% in both arms. DOR was 12.7 (95% CI: 10.5, 17.4) mo with atezo + cabo and 14.8 (95% CI: 11.3, 20.0) mo with cabo
CANTATA [45]	2	57% had received one line of therapy; 43% had received 2 lines; 62% of patients had received prior ICI therapy, in 29% it was ipilimumab plus nivolumab.	Yes	Cabo-telaglenastat vs. placebo	mPFS is not better with cabozantinib-telaglenistat. Note: ORR 41% by central review (95% CI 35–47%) for Cabozantinib monotherapy
ENTRATA [46]	2	Patients in both arms were heavily pretreated, having received a median of three prior therapies in the advanced/metastatic setting; 23% of patients were on their fifth- or later-line of therapy. 70% had received at least two prior TKIs, and 88% had prior anti-PD-(L)1 therapy.	Yes	Telaglenistat–everolimus vs. placebo-everolimus	mPFS 3.8 mo for telaglenistat–everolimus vs. 1.9 mo for placebo-everolimus. Significant
LITESPARK-005 [47]	3	Up to 3 lines of therapy, which should have included an anti-PD(L)-1 and a VEGF TKI.	Yes	Belzutifan vs. everolimus	mPFS 5.6 mo for belzutifan vs. 5.6 mo for everolimus. Significant. mOS 21.4 mo belzutifan vs. 18.1 mo with everolimus, not significant.

**Table 5:** First-Line options in metastatic Clear Cell RCC for Favorable Risk Patients are recommended by both ASCO and European Guidelines.

First-Line Treatment Options in Favorable Risk Patients
Pembrolizumab and Axitinib
Pembrolizumab and Lenvatinib
Nivolumab and Cabozantinib
Sunitinib
Pazopanib

**Table 6:** First-Line options in metastatic Clear Cell RCC for Intermediate/Poor-Risk Patients are recommended by both ASCO and European Guidelines (notwithstanding access limitations).

First-Line Treatment Options in Intermediate/Poor-Risk Patients
Ipilimumab and Nivolumab
Pembrolizumab and Axitinib
Pembrolizumab and Lenvatinib
Nivolumab and Cabozantinib
Avelumab and Axitinib

## Data Availability

The authors will provide additional information on their research. For more information, please contact the corresponding author.
